# A comparative study of effect of polyol-based and acid-based deep eutectic solvents pretreatment of rice straw under autoclave conditions on cellulose enrichment and digestibility

**DOI:** 10.1007/s13399-025-06896-9

**Published:** 2025-09-06

**Authors:** Longinus Ifeanyi Igbojionu, Jinesh Cherukkattu Manayil, Alfred Fernandez-Castane

**Affiliations:** 1 Energy and Bioproducts Research Institute (EBRI), Aston University, Aston Triangle, Birmingham, B4 7ET UK; 2 Aston Institute for Membrane Excellence (AIME), Aston University, Aston Triangle, Birmingham, B4 7ET UK

**Keywords:** Deep eutectic solvents, Rice straw, Delignification, Cellulose enrichment, Crystallinity index, Enzymatic digestibility

## Abstract

Deep eutectic solvents (DESs) are attractive for lignocellulosic biomass deconstruction because of their easy synthesis, reusability, inexpensive nature, and eco-friendliness. Pretreatment of rice straw was carried out using three types of DES: choline chloride:glycerol (ChCl:Gly), choline chloride:ethylene glycol (ChCl:EG), and choline chloride:formic acid (ChCl:FA) at 120 °C, 1.5 bars in an autoclave for 1 h. Enzymatic digestibility of pretreated samples was assessed at 10% solids loading and 9.0 filter paper units g^−1^ cellulose of enzyme loading. A delignification efficiency of 51.6% was obtained with ChCl:FA compared to 30.4% and 36.1% obtained with ChCl:Gly and ChCl:EG respectively. The cellulose content of 58.4% was obtained from ChCl:FA pretreated samples compared to cellulose contents of < 48% from ChCl:Gly and ChCl:EG pretreated samples; cellulose content of pretreated and untreated samples correlated with their crystallinity indexes. The lowest hemicellulose content of 9.5% was obtained from the ChCl:FA pretreated sample, while ChCl:Gly and ChCl:EG were like the untreated (around 22%). As indicated by spectroscopic, microscopic, and thermogravimetric analysis, DES pretreatment caused alterations in chemical composition, structure, and surface morphology. ChCl:FA pretreated sample (Ac-PT4) was significantly hydrolysed, resulting in a glucose yield of 79.7% and a concentration of 45.56 gLl^−1^ after 72 h of hydrolysis. The glucose yield obtained from ChCl:Gly and ChCl:EG pretreated samples ranged from 32 to 36%, while the glucose concentrations ranged from 14 to 16 gL^−1^. Pretreatment using ChCl:FA effectively delignified rice straw and led to a threefold increase in enzymatic digestibility compared to untreated; hence, pretreatment using ChCl:FA could support a biorefinery concept.

## Introduction

Extensive use of petroleum resources and their environmental impact as well as carbon footprints and global warming have underscored the importance of lignocellulosic biomass (LCB) as a future substitute for petroleum-based energy and chemicals. Biomass has the potential to meet the demand for clean energy and fuels for a more sustainable future [1–3]. Presently, ethanol blend mandates ranging from E10 to E20 (10–20% ethanol) have been adopted by many countries for automobiles because of their positive environmental impact [4, 5]. Currently, sugar crops such as sugarcane and sugar beet are widely used for large-scale ethanol production, thereby raising a serious concern on global food security [6, 7]. However, global attention is now focused on LCB including agricultural residues as a future replacement for petroleum to produce energy and biobased chemicals [6, 8, 9]. Additionally, LCB can sequester CO_2_ [10], thus reducing CO_2_ emissions [11]. Generally, LCB is composed of cellulose, hemicellulose, and lignin [12, 13].

The annual global production of rice is estimated to be around 980 million tons [14], which is equivalent to around 1 billion tons of straw generated annually worldwide [15]. However, rice straw is commonly burnt in the field by farmers, resulting in environmental pollution [16, 17]. Conversely, utilizing agricultural waste such as rice straw for biofuel production can serve as a sustainable energy source with concomitant benefits [14, 18, 19]. The recalcitrant nature of LCB hinders unrestricted access to the polysaccharides by cellulases [20]. Thus, the recalcitrant structure of LCB must be modified via pretreatment to facilitate its amenability to hydrolytic enzymes [21].

Several pretreatment strategies have been applied to fractionate LCB, and these can be categorized into chemical pretreatment, biological pretreatment, physical pretreatment, and the combination of any two or more of these categories [22–24]. Currently, deep eutectic solvents (DESs) have gained interest as greener solvents for LCB pretreatment [11, 25, 26]. The remarkable ability of DES to disintegrate lignin-carbohydrate interactions has contributed to the selection and deployment of several DES for the deconstruction of LCB with some promising outcomes [25, 27]. DES is produced by combining hydrogen bond acceptor and hydrogen bond donor with concomitant unique attributes such as low cost, easy to prepare, reusable, eco-friendly, and biodegradable [12, 28]. Choline chloride (ChCl) is a hydrogen bond acceptor [27], while a polyol or carboxylic acid acts as a hydrogen bond donor [12]. DES pretreatment using an acidic hydrogen bond donor such as formic acid has been extensively studied and shown to be effective for LCB delignification; hence, this DES type is used as a benchmark in the present study. However, acidic DES can degrade polysaccharide fractions and cause corrosion to equipment [29]. Therefore, to overcome these challenges, neutral polyol-based DES such as ChCl:glycerol (ChCl:Gly) and ChCl:ethylene glycol (ChCl:EG) have been investigated for the pretreatment of LCB using solids loadings in the range of 5–10% [30, 31].

The present study aimed to investigate ChCl:Gly pretreatment of rice straw under autoclave conditions at 15% solids loading. To our knowledge, ChCl:Gly pretreatment in autoclave at 15% solids loading is still missing in the literature. The efficiency of ChCl:Gly pretreatment at 10% and 15% solids loading under autoclave conditions for rice straw in terms of lignin removal and enzymatic digestibility was studied and compared with other types of DES such as ChCl:ethylene glycol and ChCl:formic acid. ChCl:Gly, ChCl:EG, and ChCl:formic acid were selected to allow a comparative study of the effects of acid and polyol-based DES on biomass delignification and enzymatic digestibility. In addition, the effect of ChCl:Gly pretreatment at 15% solids loading on biomass chemical composition, structure, and surface morphology was determined and compared with other types of DES (ChCl:EG and ChCl:FA).

## Materials and methods

### Materials and chemicals

Rice straw was obtained from a farmer in Akure, Nigeria (7.250771, 5.210266). The feedstock material was crushed with a knife mill (CM4000, LAARMANN, Roermond, Netherlands) into smaller particle sizes capable of passing through mesh 32 and 35, resulting in a particle size of around 0.5 mm. The resulting matter with a moisture content of < 10% was stored separately in plastic containers in a dry place before use. The particle size used in the present study was selected based on previous studies where particle size in the range of 0.18–0.85 mm was reported to contribute to effective delignification of LCB [32, 33]. All chemicals required were purchased from Fisher Scientific (Leicestershire, UK). Cellulase (Cellic® CTec3 HS) was kindly donated by Novozymes A/S (Bagsvaerd, Denmark).

### Preparation of DES

One molar of ChCl (hydrogen bond acceptor) was mixed with 2 molars of the respective hydrogen bond donor (glycerol, formic acid and ethylene glycol) in 200-mL glass bottles to obtain the DES. The binary mixtures (ChCl:Gly, ChCl:FA, and ChCl:EG) were allowed to react under atmospheric pressure at 70 °C for 3 h under constant stirring (250 rpm) until a homogeneous and colourless solution was obtained. Afterwards, the mixture was cooled down and kept at room temperature before use.

### DES pretreatment

The pretreatment conditions investigated are shown in Table 1. About 2.0 g and 3.0 g of raw rice straw were mixed with 20.0 g and 45.0 g of DES respectively in a 100-mL Erlenmeyer flask, corresponding to a solids loading of 10% and 15% respectively. Pretreatment was carried out in an autoclave at 120 °C, 1.5 bars for 1 h as previously described by Igbojionu et al. [21]. Subsequently, the solid and liquid phases were separated using a centrifuge (Heraeus Multifuge X1R, Thermo Scientific Inc., Madison, WI, USA) at 3900 rpm (1769 × g), 20 °C for 20 min to allow the separation of solids and liquid fractions. The solid fraction was washed twice with 10 mL of ethanol and filtered under vacuum, and this was followed by another wash with distilled water (10 mL). The sample was dried at 50 °C to a constant weight, allowed to cool down to room temperature, and stored in airtight containers until use.

**Table 1:** Type and composition of deep eutectic solvent and pretreatment condition. Rice straw was pretreated at 120 °C, 1.5 bars for 1 h in autoclave. Ac-PT1 and Ac-PT2 were pretreated at 10% and 15% solids loading respectively using ChCl:glycerol (1:2). Ac-PT3 and Ac-PT4 were pretreated at 10% and 15% solids loading respectively using ChCl:formic acid (1:2). Ac-PT5 and Ac-PT6 were pretreated at 10% and 15% solids loading respectively using ChCl:ethylene glycol

S. no	Sample name	Deep eutectic solvent	Molar ratio	Solids loading
1	Ac-PT1	ChCl:glycerol	1:2	10%
2	Ac-PT2	ChCl:glycerol	1:2	15%
3	Ac-PT3	ChCl:formic acid	1:2	10%
4	Ac-PT4	ChCl:formic acid	1:2	15%
5	Ac-PT5	ChCl:ethylene glycol	1:2	10%
6	Ac-PT6	ChCl:ethylene glycol	1:2	15%

### Analysis of the chemical composition of untreated and pretreated rice straw

Pretreated rice straw was analysed for their major constituents (cellulose, hemicellulose, lignin, and ash) by applying the methods from the National Renewable Energy Laboratory [34]. About 0.3 g of pretreated or untreated rice straw of moisture content around 10% was treated with 3 mL of 72% (w/v) H_2_SO_4_ for 1 h in a Teflon screw-capped pressure tube at 30 °C (water bath) with stirring every 10 min using a glass stirrer. Afterwards, the acid concentration was reduced to 4% (w/v) by adding 84 mL of deionized water and subsequently heated at 121 °C for 1 h in an autoclave. Then, the acid hydrolysate was neutralized using CaCO_3_ to a pH of around 5 as described by [34] and allowed to settle in a refrigerator (4 °C) before the liquid fraction was collected for sugar analysis. Monomeric sugars such as glucose, xylose, arabinose, and mannose were used to determine the corresponding polymers (cellulose and hemicellulose) based on external sugar recovery standards.

Sugar analysis was performed using HPLC (high-performance liquid chromatography) (1260 Infinity II) equipped with a Hiplex H column (Agilent) and connected to a refractive index detector. The sugar recovery standards consisting of glucose, xylose, arabinose, galactose, mannose, and acetic acid were used for making the calibration curves. The conditions were 5 mM H_2_SO_4_ of mobile phase, 0.6 mL min^−1^ of flow rate, 25 °C of column temperature, and 35 °C of detector temperature.

Klason-lignin and ash content in pretreated and untreated samples was determined as described by [34]. Acid-soluble lignin was quantified by taking the absorbance at 240 nm of the hydrolysate before neutralization using a UV–visible spectrophotometer (Evolution 220, Thermo Fisher Scientific Inc., Madison, WI, USA). [35]

Delignification efficiency was calculated as described in Eq. 1.

1 $$Delignification\;efficiency\;\left(\%\right)=\frac{\%\;of\;lignin\;\left(raw\;sample\right)-\%\;of\;lignin\;\left(pretreated\;sample\right)}{\%\;of\;lignin\;\left(raw\;sample\right)}\times100$$

### X-ray diffraction studies on pretreated rice straw

The X-ray diffraction studies were performed on untreated and pretreated rice straw using an X-ray diffractometer (D8 Advance instrument, Bruker, Germany) at a voltage and current of 40 kV and 40 mA, respectively; scanning range of 10–80° (2*θ*) was applied with a 6°/min scanning rate. The crystallinity index (CrI) was calculated using the Segal method [36].

2 $$CrI\left(\%\right)=\left(l_{002-}\;l_{am}\right)/l_{002}\times100$$

where *I*_002_ is the diffraction at 22 °C, and *I*_am_ is diffraction at 18 °C.

### Attenuated total reflectance-Fourier transform infrared (ATR-FTIR) spectroscopic studies

The untreated and pretreated biomass was studied using ATR-FTIR spectroscopy to detect the presence of different functional groups in the pretreated biomass as described by [37]. The spectra were taken at wavenumbers ranging from 400 to 4000 cm^−1^ at 4 cm^−1^ resolution and 20 scans with the aid of FTIR spectroscopy (Nicolet iS50, Thermo Fisher Scientific Inc., Madison, WI, USA) and attenuated total reflection (ATR) accessory equipped with a diamond crystal (Smart Orbit Diamond ATR, Thermo Fisher, USA).

### Surface morphology studies using scanning electron microscopy (SEM)

This analytical procedure allows a sample to be scanned with an electron beam to produce a magnified image for surface visualization. SEM analysis enables the structures of the pretreated sample to be assessed, using the method described by [37]. The JEOL JSM 7800 F PRIME (JEOL Ltd, UK) with a magnification range of × 20–100,000, while the element detection range of C-Am and acceleration voltage of 10 kV was used.

### Thermogravimetric analysis (TGA)

The Thermogravimetric Analyzer (TGA/DSC 2, Mettler-Toledo Ltd, UK) was applied to pretreated rice straw to assess the weight loss characteristics and volatile components. Nitrogen (carrier gas) with a flow rate of 70 mL/min was used in the TGA. About 3.0 mg of pretreated rice straw was weighed into a clean crucible and transferred to the sample holding chamber. The heating program was set to 40 to 400 °C and the heating rate was 10 °C/min. OriginPro software was employed to plot both thermogravimetric and differential thermogravimetric curves.

### Enzymatic hydrolysis

Cellic® CTec3 HS was employed for the enzymatic hydrolysis. The enzyme activity (74 FPU mL^−1^) was determined following a standard method [38]. The hydrolysis was carried out in a 15-mL screw-capped glass tube at 10% solids loading. 1.0 g of sample was suspended into 10 mL of 0.2 M phosphate buffer (pH 5.0) with enzyme loading of 9.0 FPU g^−1^ cellulose. Thereafter, 0.5 g L^−1^ sodium azide was added to prevent contamination and microbial growth. The tube was placed in an incubator (Incu-Shake MAXI, SciQuip Ltd, Rotherham, UK) at 50 °C with constant shaking (200 rpm) for 72 h. The sample was collected at 24-h intervals and the enzyme was deactivated by incubating in a water bath at 100 °C for 5 min. All tests were carried out in duplicate. The sugar in the aliquots was determined by HPLC as described in Section 2.4, and glucose yield was calculated using Eq. 3.

3 $$Glucose\;yield\left(\frac{Total\;amount\;of\;glucose\times0.9}{Total\;mount\;if\;Cellulose}\right)\times100$$

## Results and discussion

### Effects of DES pretreatment on chemical composition

**Fig. 1:**
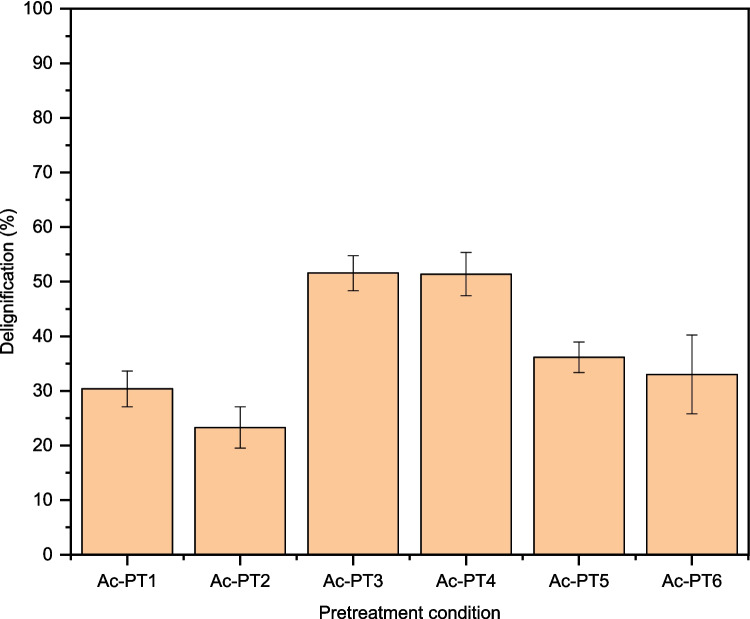
Delignification efficiency of ChCl:Gly, ChCl:EG, and ChCl:FA on rice straw pretreated under autoclave conditions. Average values are presented, and the error bars correspond to standard deviation

ChCl-based DES can break the linkages within lignin carbohydrate complex in the LCB [39, 40], resulting in lignin solubilization and removal. Three types of DESs, namely, ChCl:Gly, ChCl:EG, and ChCl:FA, were prepared, and their effectiveness in the delignification of rice straw at mild conditions was evaluated. Delignification efficiencies of the three types of DES on rice straw are shown in Fig. 1. ChCl:FA pretreated samples (Ac-PT3 and Ac-PT4) exhibited delignification efficiency ranging from 51.4 to 51.6%, while the delignification efficiency of ChCl:Gly (Ac-PT1 and Ac-PT2) and ChCl:EG (Ac-PT5 and Ac-PT6) pretreated samples ranged from around 23.3 to 36.2%. However, increases in the solid loading from 10 to 15% did not significantly affect the delignification efficiency of ChCl:FA as the efficiency decreased slightly to 51.4%, whereas the delignification efficiency of ChCl:Gly and ChCl:EG decreased significantly to 23.0% and 33.0% respectively with increases in solid loading rate. ChCl:FA exhibited the highest delignification efficiency compared to ChCl:Gly and ChCl:EG, which displayed relatively lower delignification efficiency.

The low delignification efficiency of ChCl:Gly and ChCl:EG may be attributed to high viscosity which could hinder their ability to penetrate the biomass structure [41]. On the contrary, ChCl:FA with lower viscosity could have a better ability to penetrate the biomass structure and break the linkages within the lignin carbohydrate complex resulting in lignin solubilization and removal [32]. The delignification efficiency of 51.6% obtained using ChCl:FA was higher than the 44% delignification efficiency reported by other authors from rice straw [42]. Secondly, 33.5% and 20.6% of lignin removal from oil palm empty fruit bunch were reported after pretreatment using ChCl:lactic acid (1:1) and ChCl:citric acid (1:1) respectively [43].

**Fig. 2:**
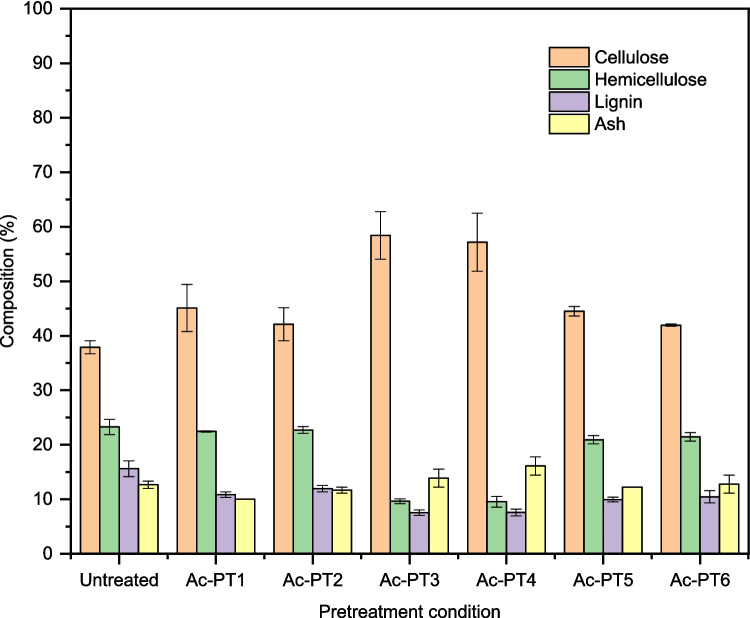
Proximate composition of rice straw solid residues obtained after pretreatment in autoclave using ChCl:Gly, ChCl:EG, and ChCl:FA respectively. Average values are presented, and the error bars correspond to standard deviation

Figure 2 shows the composition of rice straw pretreated using three types of DES (ChCl:Gly, ChCl:EG, and ChCl:FA). The untreated rice straw comprised 37.9% cellulose, 23.3% hemicellulose, 15.6% lignin, and 12.7% ash. According to the report in the literature, rice straw is comprised of 42.9 wt % cellulose, 23.9 wt % hemicellulose, 16.7 wt % lignin, and 11.5 wt % ash (Aggarwal et al. 2021). After DES pretreatment, the cellulosic content of samples increased from 42.1 to 58.4%. The highest increase in the cellulosic content (58.4%) was obtained from rice straw pretreated with ChCl:FA (Ac-PT3) while the lowest content (42.0%) was obtained from rice straw pretreated with ChCl:EG (Ac-PT6). Generally, ChCl:FA pretreated samples showed high cellulosic content compared to ChCl:Gly and ChCl:EG pretreated samples, which can be attributed to high lignin solubilization and removal during ChCl:FA pretreatment. Other authors reported cellulose contents ranging from 35.9 to 58.4% from rice straw pretreated with different types of DES at a temperature similar to that used in the present study, but the reaction time was six times higher [44].

However, hemicellulose contents of around 10% were obtained from Ac-PT3 and Ac-PT4, respectively, while hemicellulose contents of more than 20% were obtained from both ChCl:Gly (Ac-PT1 and Ac-PT2) and ChCl:EG (Ac-PT5 and Ac-PT6) pretreated samples. The hemicellulose content of ChCl:Gly and ChCl:EG pretreated samples was similar to the untreated, suggesting that pretreatment using these types of DES did not cause hemicellulose solubilization and removal. On the other hand, ChCl:FA pretreatment resulted in significant decreases in hemicellulose content, which can be attributed to hemicellulose solubilization. Other authors reported significant decreases in hemicellulose fraction from rice straw pretreated using acidic DES such as ChCl:formic acid and ChCl:acetic acid at 130 °C [42].

The lignin content of pretreated rice straw ranged from 7.6 to 12.0%. The highest lignin content was obtained from Ac-PT2 (12.0%), while the lowest lignin content of around 7.6% was obtained from Ac-PT3 and Ac-PT4. The relatively low amount of lignin and hemicellulose in the ChCl:FA pretreated samples could be attributed to bond cleavage catalysed by protons in the acidic DES [45]. Secondly, monocarboxylic acids such as formic acid with shorter alky chain length were reported to show stronger lignin solubility [46], thus confirming the low lignin content of ChCl:FA pretreated samples due to lignin solubilization and removal. Maibam et al. [47] reported a lignin content of 7.9% from rice straw pretreated with acidic DES (ChCl:Acetic acid), which was higher than the value of 7.6% obtained with ChCl:FA pretreated sample (Ac-PT3) in the present study. The ash content of pretreated samples ranged from 10.0 to 16.1% compared with 12.7% in the untreated. The highest ash content was obtained from Ac-PT4 (16.1%), while the least ash content of 10.0% was obtained from Ac-PT1. The high ash content in ChCl:FA pretreated samples could be attributed to the effect of the high severity of the pretreatment condition due to very low pH (≈1).

### Effect of DES pretreatment on ultrastructure

**Fig. 3:**
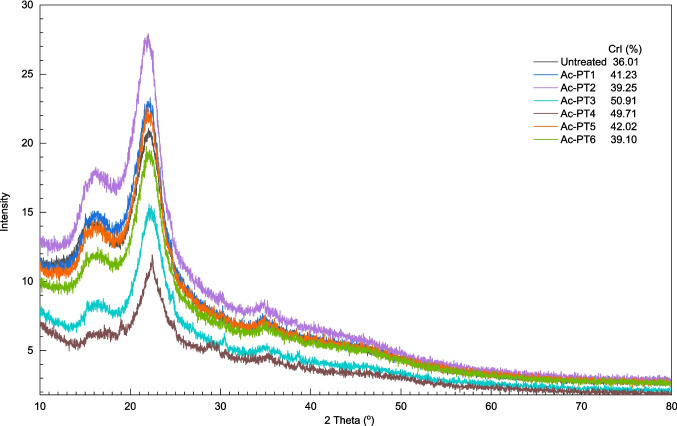
XRD spectra of untreated and pretreated rice straw using ChCl:Gly, ChCl:EG, and ChCl:FA respectively

Rice straw consists of cellulose, hemicellulose, and lignin which are complex and heterogenous. The crystalline structure of cellulose necessitates pretreatment to enhance its amenability to enzymatic attack [48]. Thus, the crystallinity of cellulose is considered a major factor affecting enzymatic saccharification because crystalline cellulose is more resistant to attack by enzymes than amorphous cellulose [48–50]. XRD was performed on DES pretreated samples to gain insight into the impact of the selected pretreatment methodologies. Figure 3 shows the XRD spectra and CrI of untreated and pretreated samples. A narrower and more precise peak of the crystal plane was observed in the pretreated samples compared to a broader peak from the untreated rice straw. Furthermore, the intensity of peaks became stronger in the pretreated samples, suggesting decreases in the amorphous part of the biomass and increases in the crystalline proportion of cellulose. Nevertheless, the located position of the peaks did not change, and no additional peaks were observed, thus suggesting that the original crystal structure of cellulose remained unchanged after pretreatment.

CrI of pretreated samples increased from 39.4% for ChCl:Gly pretreated sample (Ac-PT2) to 50.9% for ChCl:FA pretreated (Ac-PT3) compared to untreated (36.0%). Samples pretreated using ChCl:Gly (Ac-PT1 and Ac-PT2) and ChCl:EG (Ac-PT5 and Ac-PT6) exhibited lower CrIs, while samples pretreated with ChCl:FA (Ac-PT3 and Ac-PT4) exhibited higher CrI values. However, increasing the biomass loading rate from 10 to 15% resulted in slight decreases in CrI of pretreated samples. In ChCl:Gly pretreated samples, CrI decreased from 41.2% (Ac-PT1) to 39.3% (Ac-PT2) with an increase in solid loading, while CrI decreased from 50.9% (Ac-PT3) to 49.7% (Ac-PT4) in ChCl:FA pretreated samples. Similarly, the increase in solid loading from 10 to 15% decreased the CrI of ChCl:EG pretreated samples from 42.0% (Ac-PT5) to 39.1% (Ac-PT6). The lack of significant changes in the CrI indicates a less substantial alteration in the crystalline and amorphous regions of the cellulosic fractions. However, the differences in CrI between ChCl:FA and other DES types (ChCl:Gly and ChCl:EG) are mainly due to differences in their cellulose contents as CrI appeared to correlate with cellulose content of pretreated samples. The relatively lower CrI obtained with samples pretreated using ChCl:Gly and ChCl:EG may be connected to their neutral pHs and high viscosities, resulting in lower lignin solubilization and removal [28]. On the contrary, the higher CrI obtained from ChCl:FA pretreated samples indicates higher lignin solubilization and removal, which may be connected to the low viscosity and acidic pH of ChCl:FA.

The higher CrI values obtained in all the pretreated samples compared to the untreated are mainly due to crystallographic domains being organized into more ordered states because of the solubilization and removal of lignin [51, 52]. Secondly, increased CrI in pretreated samples is connected to removing lignin, amorphous hemicellulose, and part of amorphous cellulose [53].

### Effect of DES pretreatment on the chemical structure

**Fig. 4:**
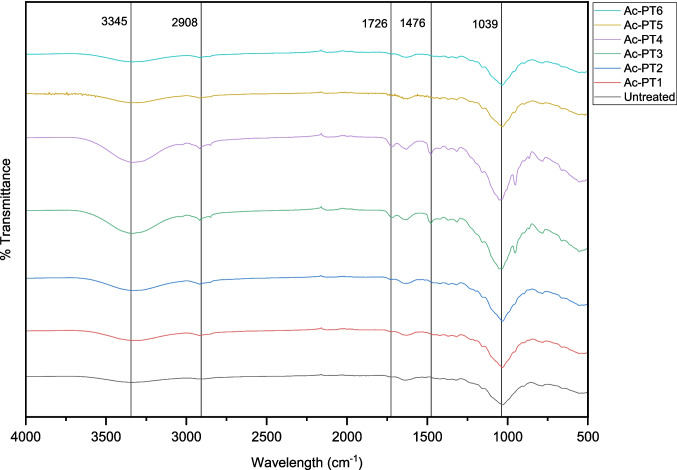
ATR-FTIR spectra of untreated and pretreated rice straw using ChCl:Gly, ChCl:EG, and ChCl:FA respectively

ATR-FTIR analysis was performed to gain insights into the effects of DES pretreatment on the chemical structure of pretreated and untreated rice straw, as shown in Fig. 4. The peaks located at 3345 cm^−1^ and 2908 cm^−1^ represent the distinguished peaks of cellulose, especially O–H stretching and C-H stretching [54]. These bands were broader in ChCl:FA pretreated samples (Ac-PT3 and Ac-PT4) indicating the high cellulose content of the samples. The observed changes in the band at 2908 cm^−1^ indicated that some rupture might have occurred in the methyl and methylene groups of cellulose. However, the broad peak observed at 1039 cm^−1^ was typically related to the structural characteristics of cellulose and hemicellulose [54, 55]. The strengths of these peaks were higher in ChCl:FA pretreated samples compared to ChCl:Gly and ChCl:EG pretreated samples due to high lignin removal during ChCl:FA pretreatment, resulting in increases in cellulose content. On the other hand, the observed peaks at 1726 cm^−1^ and 1476 cm^−1^ correspond to lignin removal respectively [54]. These peaks occurred in ChCl:FA pretreated samples, indicating some structural changes in the biomass due to lignin removal. However, these peaks were absent in ChCl:Gly and ChCl:EG pretreated samples, suggesting no structural change occurred due to the poor delignification efficiency of ChCl:Gly and ChCl:EG.

### Effect of DES pretreatment on surface morphology

**Fig. 5:**
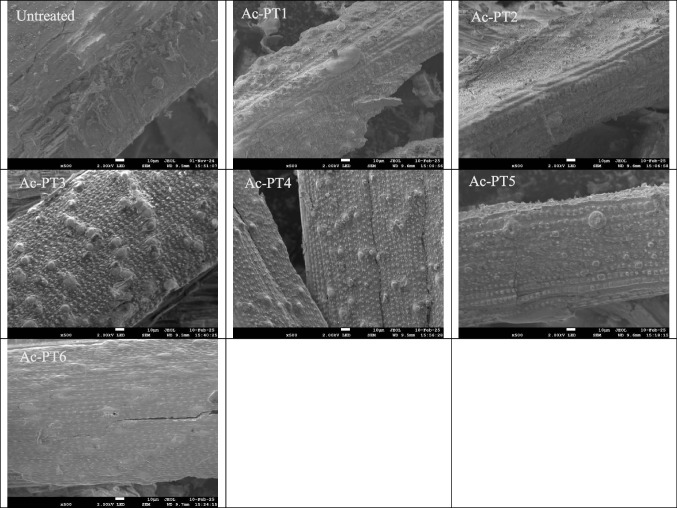
SEM images of untreated and pretreated samples (Ac-PT1, Ac-PT2, Ac-PT3, Ac-PT4, Ac-PT5, and Ac-PT6)

SEM was carried out to understand better the effect of DES pretreatment on the surface morphology of rice straw, including fiber destruction and increased porous structure [56]. Figure 5 shows the surface morphology of rice straw after DES pretreatment was applied. The untreated biomass had a smooth intact structure featuring an ordered fibrous structure before pretreatment. This non-porous and rigid structure can hinder cellulose accessibility to hydrolytic enzymes [57]. In ChCl-Gly pretreated samples (Ac-PT1 and Ac-PT2), a slightly rough surface was observed with some patches, while ChCl-EG pretreated samples (Ac-PT5 and Ac-PT6) displayed a relatively rough surface and somewhat perforated. In general, the morphology of ChCl-Gly and ChCl:EG pretreated samples appeared not to be seriously damaged, which could be attributed to less severity of the pretreatment condition characterized by low lignin removal.

On the other hand, ChCl-FA pretreated samples (Ac-PT3 and Ac-PT4) had a rough surface with large perforations. This may be due to the high removal of lignin and hemicellulose fractions, resulting in free and disorganized dense fiber bundles. Thus, as the amount of lignin and xylan removed from the sample increased, more of the surface area became exposed and fibrils disordered, with a concomitant increase in cellulose accessibility by the hydrolytic enzyme [25, 56]. Also, the dot-like structures observed in ChCl-FA pretreated samples (Ac-PT3 and Ac-PT4) and ChCl-EG pretreated samples (Ac-PT5) could indicate pseudo-lignin formation via lignin condensation due to the harsh pretreatment condition [58] (Chen et al., 2024).

### Effect of DES pretreatment on thermal degradation

**Fig. 6:**
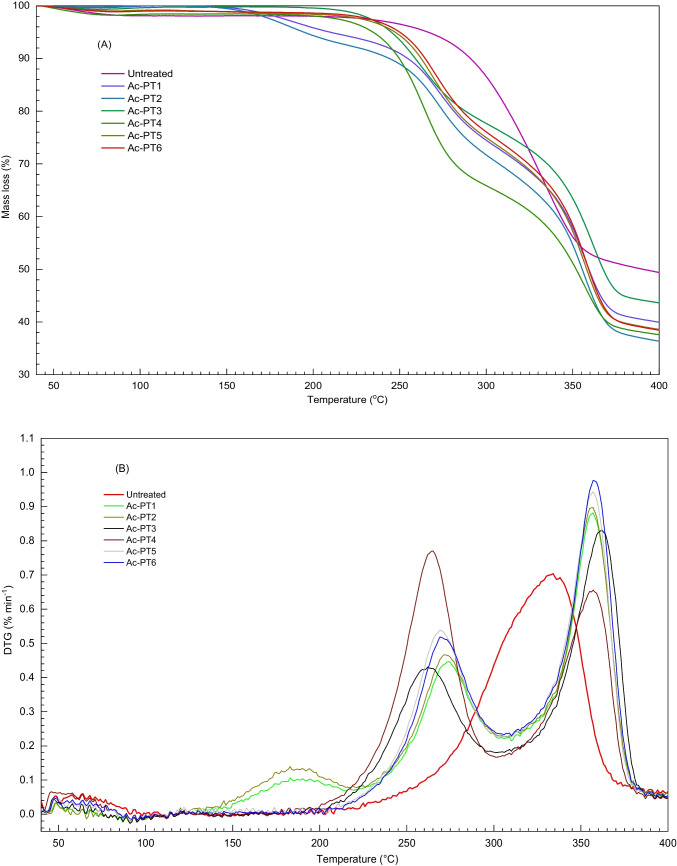
Thermal analysis of untreated and pretreated rice straw. **A** Thermogravimetric analysis (TGA) curves. **B** Differential thermogravimetric (DTG) curves

The thermal behaviour of DES pretreated samples was determined through TGA and DTG analysis, as shown in Fig. 6. The TGA profiles (Fig. 6A) of pretreated samples exhibited different patterns due to variations in their chemical compositions. All samples exhibited similarity in their TGA profiles from 50 to 150 °C, and thereafter, the profiles of pretreated samples started to deviate from each other due to the different components in the samples. This suggests that DES pretreatment may have caused structural changes, hence the observed difference in the degradation path. In a typical pyrolysis process, three stages through which mass loss occurs are moisture evaporation (50–150 °C), fast devolatilization (150–400 °C), and carbonization (450–650 °C) [59]. In the first stage, moisture evaporation occurs, including the release of volatile CO and CO_2_. In the second stage, hemicellulose degradation occurs at temperatures around 200–350 °C, while cellulose and lignin degradation occur at temperatures around 350–400 °C resulting in a large percentage of mass loss. In the carbonization stage, mass loss gradually decreases and becomes almost flattened in the TG-DTG curves [59].

The DTG curves (Fig. 6B) for the pretreated biomasses showed a distinctive thermal degradation behaviour compared to that of the untreated. Unlike the untreated samples, two degradation peaks were obtained for the pretreated samples, where a single broad peak occurred. The peak obtained at around 190 °C for ChCl:Gly pretreated samples may be due to the degradation of residual glycerol in the samples. Other authors reported degradation temperatures of 195–218 °C and 180–212 °C for crude and pure glycerol [60]. The peak around 250–270 °C in the pretreated samples indicates hemicellulose decomposition. However, the highest mass loss of 0.8% was obtained with Ac-PT4 while Ac-PT3 was the least (0.4%). The second degradation peak obtained around 350–380 °C for the pretreated samples indicates decomposition of hemicellulose and cellulose, and a part of lignin. The lower mass loss obtained in ChCl:FA pretreated samples compared to ChCl:Gly and ChCl:EG pretreated samples indicates crystalline cellulose resistant to decomposition at this temperature. This finding was consistent with the report of other authors, where the crystalline structure of LCB increases the thermal degradation temperature of cellulose [61]. The broad peak obtained around 300–360 °C in untreated rice straw indicates that hemicellulose, cellulose, and part of lignin were decomposed within this temperature range. Dev et al. [62] reported a thermal degradation temperature of 250–365 °C for hemicellulose and cellulose degradation and 300–700 °C for lignin from pretreated rice straw. Other authors reported thermal degradation of 230–450 °C for cellulose, 180–340 °C for hemicellulose, and over 500 °C for lignin [59].

### Effect of DES pretreatment on biomass digestibility

**Fig. 7:**
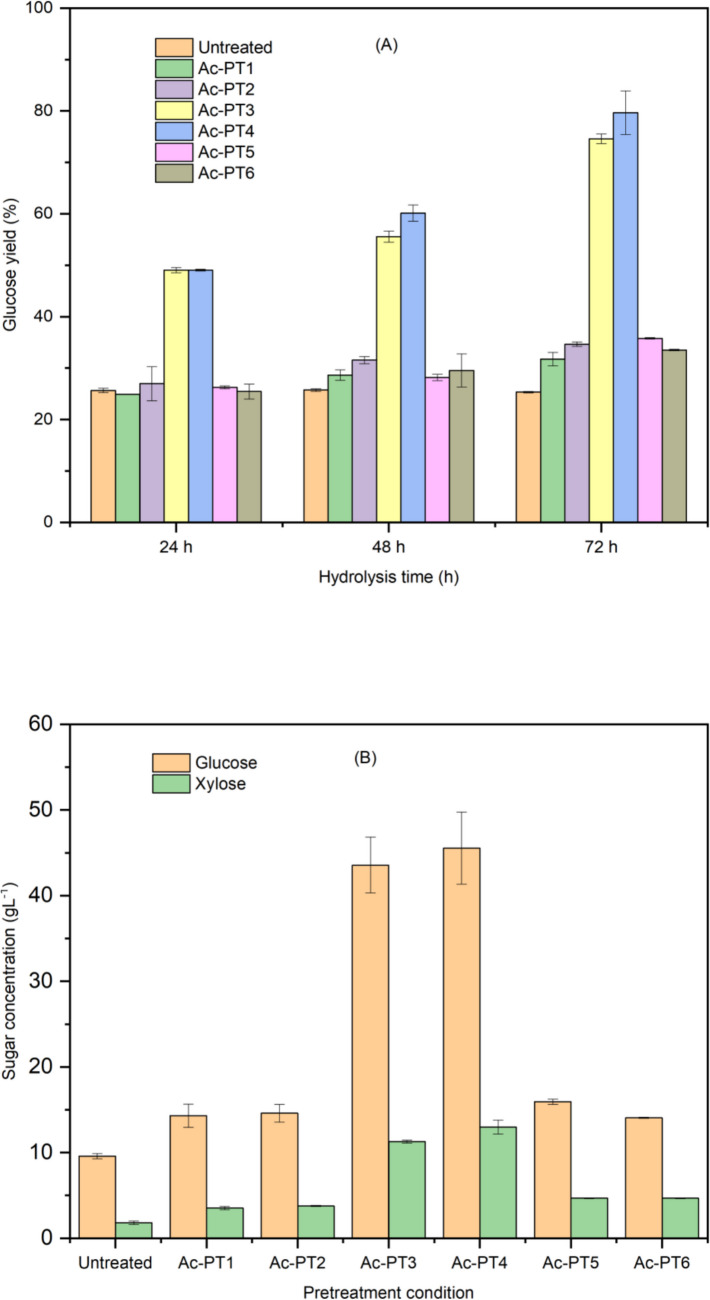
Enzymatic hydrolysis of untreated and pretreated rice straw. **A** Glucose yield from untreated and pretreated rice straw. **B** Glucose and xylose concentration after 72 h. Average values are presented, and the error bars correspond to standard deviation

Enzymatic hydrolysis was performed on the DES pretreated samples, and the efficiency of enzymatic digestibility of pretreated samples was determined and evaluated based on the amounts of sugars (glucose and xylose) released. The result of enzymatic hydrolysis (yield of glucose and sugars concentration) is depicted in Fig. 7. After 24 h, the glucose yield from untreated rice straw was 25.7%; this value was similar to ChCl:Gly (Ac-PT1 and Ac-PT2) and ChCl:EG (Ac-PT5 and Ac-PT6) pretreated samples (Fig. 7A). The low glucose yields may be due to the ineffectiveness of ChCl:Gly and ChCl:EG pretreatment in altering the recalcitrant structure of biomass via lignin solubilization [29]. However, glucose yields of 49.1% and 49.2% were obtained for Ac-PT3 and Ac-PT4 respectively after 24 h. After 48 h of hydrolysis, glucose yield remained unchanged for the untreated, but the glucose yield for ChCl:Gly and ChCl:EG pretreated samples increased slightly to around 29%. However, glucose yields of 55.6% and 60.1% were obtained for Ac-PT3 and Ac-PT4 respectively after 48 h. Glucose yields for Ac-PT3 and Ac-PT4 increased to 74.6% and 79.7% respectively after 72 h. On the contrary, the glucose yield from untreated samples remained relatively unchanged (25.3%) after 72 h, whereas the glucose yields for ChCl:Gly and ChCl:EG pretreated samples increased to values ranging from around 32 to 36%. This low digestibility suggests that pretreatment using ChCl:Gly and ChCl:EG was inefficient in the delignification of rice straw, resulting in decreased accessibility to cellulose by the hydrolytic enzyme due to unproductive adsorption of cellulase on lignin by binding with lignin [20].

In addition, the low glucose yield obtained from ChCl:Gly pretreated samples may be connected to the presence of residual glycerol as indicated in the TGA/DTG data. Hou et al. [44] reported a glucose yield of 30.2% and 33.9% from rice straw pretreated using ChCl:Gly and ChCl:EG, respectively, at 120 °C for 3 h, while other authors reported 36% glucose yield from rice straw pretreated with ChCl:lactic acid at 60 °C for 12 h [54]. These values reported were lower than those obtained in the present study, indicating that the DES pretreatment improved enzymatic digestibility of rice straw.

Furthermore, sugar concentration released after 72 h is shown in Fig. 7B. Glucose concentrations of 43.56 gL^−1^ and 45.56 gLl^−1^ of glucose were obtained for Ac-PT3 and Ac-PT4, respectively, while their xylose concentrations were around 13 gL^−1^ (Fig. 7B). For the untreated sample, glucose and xylose concentrations were around 9.58 gL^−1^ and 2 gL^−1^ respectively. Glucose concentration of around 14.30 gL^−1^ was obtained for Ac-PT1 and Ac-PT2, whereas glucose concentrations of 15.83 gL^−1^ and 14.05 gL^−1^ were obtained for Ac-PT5 and Ac-PT6 respectively. Xylose concentration of around 5 gL^−1^ were obtained for Ac-PT5 and Ac-PT6, which was similar to that of xylose of Ac-PT1 and Ac-PT2 (around 4 gL^−1^). The similarities in the glucose yield and sugar concentrations obtained for ChCl:Gly and ChCl:EG pretreated samples could be attributed to their chemical composition. Therefore, the glucose concentration of 45.54 gL^−1^ obtained from ChCl:FA pretreated rice straw (Ac-PT4) in the present study was significantly higher than previously reported [14, 63]. Thus, pretreatment using ChCl:FA under autoclave condition has the potential to support biorefinery concept for bioethanol production.

## Conclusion

ChCl:FA pretreatment effectively removed lignin from rice straw compared to ChCl:Gly and ChCl:EG. In addition, pretreatment using ChCl:FA led to an appreciable increase in cellulosic content (58.4%) compared to the untreated (37.9%). The changes in the chemical composition of rice straw due to pretreatment correlated with changes in its ultrastructure, chemical structure, and morphology. Pretreatment using ChCl:FA led to around 80% glucose yield, about three times more than the untreated sample. ChCl:Gly pretreatment led to low delignification efficiency and a low glucose yield of around 35%. However, ChCl:Gly and ChCl:EG could be promising solvents for biomass pretreatment at high solids loading (15%), enabling higher conversion rates. Pretreatment conditions need to be optimized to achieve more excellent lignin removal. The effect of recycling and reusing DES on lignin removal requires further studies, including its impact on the physicochemical properties of the recovered lignin.

## References

[1] Sun Q, Chen WJ, Pang B, Sun Z, Lam SS, Sonne C, Yuan TQ (2021) Ultrastructural change in lignocellulosic biomass during hydrothermal pretreatment. Bioresour Technol 341:125807. 10.1016/j.biortech.2021.125807 34474237 10.1016/j.biortech.2021.125807

[2] Kumar S, Paritosh K, Pareek N, Chawade A, Vivekanand V (2018) De-construction of major Indian cereal crop residues through chemical pretreatment for improved biogas production: an overview. Renew Sustain Energy Rev 90:160–170. 10.1016/j.rser.2018.03.071

[3] Huber GW, Iborra S, Corma A (2006) Synthesis of transportation fuels from biomass: chemistry, catalysts, and engineering. Chem Rev 106(9):4044–4098. 10.1021/cr068360d 16967928 10.1021/cr068360d

[4] Barua S, Sahu D, Sultana F, Baruah S, Mahapatra S (2023) Bioethanol, internal combustion engines and the development of zero-waste biorefineries: an approach towards sustainable motor spirit. RSC Sustain 1(5):1065–1084. 10.1039/D3SU00080J

[5] Khuong LS, Masjuki HH, Zulkifli NWM, Mohamad EN, Kalam MA, Alabdulkarem A, Jamshaid M (2017) Effect of gasoline-bioethanol blends on the properties and lubrication characteristics of commercial engine oil. RSC Adv 7(25):15005–15019. 10.1039/C7RA00357A

[6] Broda M, Yelle DJ, Serwanska K (2022) Bioethanol production from lignocellulosic biomass—challenges and solutions. Molecules 27(24):8717. 10.3390/molecules27248717 36557852 10.3390/molecules27248717 PMC9785513

[7] Mariano APB, Unpaprom Y, Ramaraj R (2020) Hydrothermal pretreatment and acid hydrolysis of coconut pulp residue for fermentable sugar production. Food Bioprod Process 122:31–40. 10.1002/er.5544

[8] Chandel AK, Garlapati VK, Singh AK, Antunes FAF, da Silva SS (2018) The path forward for lignocellulose biorefineries: bottlenecks, solutions, and perspective on commercialization. Bioresour Technol 264:370–381. 10.1016/j.biortech.2018.06.004 29960825 10.1016/j.biortech.2018.06.004

[9] Vu PT, Unpaprom Y, Ramaraj R (2018) Impact and significance of alkaline-oxidant pretreatment on the enzymatic digestibility of *Sphenoclea zeylanica* for bioethanol production. Bioresour Technol 247:125–130. 10.1016/j.biortech.2017.09.012 28946085 10.1016/j.biortech.2017.09.012

[10] Enda TF, Karaosmanoglu F (2021) Supply chain network carbon footprint of forest biomass to biorefinery. J Sustain For 40:124–141. 10.1080/10549811.2020.1746349

[11] Arce C, Llano T, Mowinckel Á, Coz A (2024) Deep eutectic solvents as pretreatment to increase Fock’s reactivity under optimum conditions. Cellulose 31(1):247–261. 10.21203/rs.3.rs-2776324/v1

[12] Provost V, Dumarcay S, Ziegler-Devin I, Boltoeva M, Trébouet D, Villain-Gambier M (2022) Deep eutectic solvent pretreatment of biomass: influence of hydrogen bond donor and temperature on lignin extraction with high β-O-4 content. Bioresour Technol 349:126837. 10.1016/j.biortech.2022.126837 35150854 10.1016/j.biortech.2022.126837

[13] Manmai N, Unpaprom Y, Ramaraj R (2021) Bioethanol production from sunflower stalk: application of chemical and biological pretreatments by response surface methodology (RSM). Biomass Convers Biorefin 11:1759–1773. 10.1007/s13399-020-00602-7

[14] Momayez F, Karimi K, Karimi S, Horváth IS (2017) Efficient hydrolysis and ethanol production from rice straw by pretreatment with organic acids and effluent of biogas plant. RSC Adv 7(80):50537–50545. 10.1039/C7RA10063A

[15] International Rice Research Institute (IRRI) The value of sustainable rice straw management. https://www.irri.org/rice-straw-management. Accessed 10 Oct 2024

[16] Islam M, Saini P, Das R, Shekhar S, Sinha A, Prasad K (2023) Rice straw as a source of nanocellulose for sustainable food packaging materials: a review. BioResources 18(1):2351. 10.15376/biores.18.1.Islam.

[17] Singh Y, Sharma S, Kumar U, Sihag P, Balyan P, Singh KP, Dhankher OP (2023) Strategies for economic utilization of rice straw residues into value-added by-products and prevention of environmental pollution. Sci Total Environ 167714:167714. 10.1016/j.scitotenv.2023.167714 10.1016/j.scitotenv.2023.167714 37832665

[18] Mujtaba M, Fraceto LF, Fazeli M, Mukherjee S, Savassa SM, de Medeiros GA, Vilaplana F (2023) Lignocellulosic biomass from agricultural waste to the circular economy: a review with focus on biofuels, biocomposites and bioplastics. J Clean Prod 402:136815. 10.1016/j.jclepro.2023.136815

[19] Kapoor M, Soam S, Agrawal R, Gupta RP, Tuli DK, Kumar R (2017) Pilot scale dilute acid pretreatment of rice straw and fermentable sugar recovery at high solid loadings. Bioresour Technol 224:688–693. 10.1016/j.biortech.2016.11.032 27864133 10.1016/j.biortech.2016.11.032

[20] Yuan Y, Jiang B, Chen H, Wu W, Wu S, Jin Y, Xiao H (2021) Recent advances in understanding the effects of lignin structural characteristics on enzymatic hydrolysis. Biotechnol Biofuels 14:1–20. 10.1186/s13068-021-02054-1 34670604 10.1186/s13068-021-02054-1 PMC8527784

[21] Igbojionu LI, Laluce C, Pecoraro E (2020) Two-stage alkaline and acid pretreatment applied to sugarcane bagasse to enrich the cellulosic fraction and improve enzymatic digestibility. Detritus 13:106–113. 10.31025/2611-4135/2020.14005

[22] Hierro-Iglesias C, Fatokun CO, Chimphango A, Bayitse R, Blanco-Sanchez PH, Thornley P, Fernandez-Castane A (2024) Process integration for efficient conversion of cassava peel waste into polyhydroxyalkanoates. J Environ Chem Eng 12(1):111815. 10.1016/j.jece.2023.111815

[23] Ferdeș M, Dincă MN, Moiceanu G, Zăbavă BȘ, Paraschiv G (2020) Microorganisms and enzymes used in the biological pretreatment of the substrate to enhance biogas production: a review. Sustainability 12(17):7205. 10.3390/su12177205

[24] Sharma HK, Xu C, Qin W (2019) Biological pretreatment of lignocellulosic biomass for biofuels and bioproducts: an overview. Waste Biomass Valoriz 10:235–251. 10.1007/s12649-017-0059-y

[25] Liu Y, Deak N, Wang Z, Yu H, Hameleers L, Jurak E, Barta K (2021) Tunable and functional deep eutectic solvents for lignocellulose valorization. Nat Commun 12(1):5424. 10.1038/s41467-021-25117-1 34521828 10.1038/s41467-021-25117-1 PMC8440657

[26] Ai B, Li W, Woomer J, Li M, Pu Y, Sheng Z, Shi J (2020) Natural deep eutectic solvent mediated extrusion for continuous high-solid pretreatment of lignocellulosic biomass. Green Chem 22(19):6372–6383. 10.1039/D0GC01560A

[27] Chen Z, Bai X, Lusi A, Zhang H, Wan C (2020) Insights into structural changes of lignin toward tailored properties during deep eutectic solvent pretreatment. ACS Sustain Chem Eng 8:9783–9793. 10.1021/acssuschemeng.0c01361

[28] Hossain MA, Rahaman MS, Yelle D, Shang H, Sun Z, Renneckar S, Sathitsuksanoh N (2021) Effects of polyol-based deep eutectic solvents on the efficiency of rice straw enzymatic hydrolysis. Ind Crops Prod 167:113480. 10.1016/j.indcrop.2021.113480

[29] del Mar C-Gámez M, Galán-Martín Á, Seixas N, da Costa Lopes AM, Silvestre A, Castro E (2023) Deep eutectic solvents for improved biomass pretreatment: current status and future prospective towards sustainable processes. Bioresour Technol 369:128396. 10.1016/j.biortech.2022.128396 10.1016/j.biortech.2022.128396 36503832

[30] Li H, Li X, Li D, Zhang J, Nawaz H, You T, Xu F (2022) Highly-efficient pretreatment using alkaline enhanced aqueous deep eutectic solvent to unlock poplar for high yield of fermentable sugars: synergistic removal of lignin and mannan. Bioresour Technol 351:126993. 10.1016/j.biortech.2022.126993 35288268 10.1016/j.biortech.2022.126993

[31] Hassan ESRE, Mutelet F (2022) Evaluation of miscanthus pretreatment effect by choline chloride based deep eutectic solvents on bioethanol production. Bioresour Technol 345:126460. 10.1016/j.biortech.2021.126460 34863844 10.1016/j.biortech.2021.126460

[32] Xu H, Peng J, Kong Y, Liu Y, Su Z, Li B, Tian W (2020) Key process parameters for deep eutectic solvents pretreatment of lignocellulosic biomass materials: a review. Bioresour Technol 310:123416. 10.1016/j.biortech.2020.123416 32334906 10.1016/j.biortech.2020.123416

[33] Chambon CL, Verdía P, Fennell PS, Hallett JP (2021) Process intensification of the ionoSolv pretreatment: effects of biomass loading, particle size and scale-up from 10 mL to 1 L. Sci Rep 11:15383. 10.1038/s41598-021-94772-w 34321510 10.1038/s41598-021-94629-z PMC8319198

[34] Sluiter A, Hames B, Ruiz R, Scarlata C, Sluiter J, Templeton D, Crocker D (2012) Determination of structural carbohydrates and lignin in biomass. Lab Anal Proced (LAP). https://www.nrel.gov/docs/gen/fy13/42618.pdf. Accessed 13 Mar 2024

[35] Poy H, Lladosa E, Arcís A, Gabaldón C, Loras S (2023) Microwave-assisted ternary deep eutectic solvent pretreatment for improved rice straw saccharification under mild pretreatment conditions. Ind Crops Prod 206:117639. 10.1016/j.indcrop.2023.117639

[36] Segal L, Creely J, Martin A, Conrad C (1959) An empirical method for estimating the degree of crystallinity of native cellulose using the X-ray diffractometer. Text Res J 29:786–794. 10.1177/004051755902901003

[37] Awoyale AA, Lokhat D (2021) Experimental determination of the effects of pretreatment on selected Nigerian lignocellulosic biomass in bioethanol production. Sci Rep 11:557. 10.1038/s41598-020-78105-8 33436682 10.1038/s41598-020-78105-8 PMC7804122

[38] Ghose TK (1987) Measurement of cellulase activities. Pure Appl Chem 59(2):257–268. 10.1351/pac198759020257

[39] Cui P, Ye Z, Chai M, Yuan J, Xiong Y, Yang H, Yao L (2023) Effective fractionation of lignocellulose components and lignin valorization by combination of deep eutectic solvent with ethanol. Front Bioeng Biotechnol 10:1115469. 10.3389/fbioe.2022.1115469 36698646 10.3389/fbioe.2022.1115469 PMC9869112

[40] Zhou X, Huang T, Liu J, Gao H, Bian H, Wang R et al (2021) Recyclable deep eutectic solvent coupling sodium hydroxide post-treatment for boosting woody/herbaceous biomass conversion at mild condition. Bioresour Technol 320:124327. 10.1016/j.biortech.2020.124327 33157438 10.1016/j.biortech.2020.124327

[41] New EK, Wu TY, Lee C, Poon ZY, Loow YL, Foo LYW, Procentese A, Siow LF, Teoh WH, Daud NNN, Jahim JM, Mohammad AW (2019) Potential use of pure and diluted choline chloride-based deep eutectic solvent in delignification of oil palm fronds. Process Saf Environ Prot 123:190–198. 10.1016/j.psep.2018.11.015

[42] Xing W, Xu G, Dong J, Han R, Ni Y (2018) Novel dihydrogen-bonding deep eutectic solvents: pretreatment of rice straw for butanol fermentation featuring enzyme recycling and high solvent yield. Chem Eng J 333:712–720. 10.1016/j.cej.2017.09.176

[43] Tan YT, Ngoh GC, Chua ASM (2019) Effect of functional groups in acid constituent of deep eutectic solvent for extraction of reactive lignin. Bioresour Technol 281:359–366. 10.1016/j.biortech.2019.02.010 30831515 10.1016/j.biortech.2019.02.010

[44] Hou XD, Li AL, Lin KP, Wang YY, Kuang ZY, Cao SL (2018) Insight into the structure-function relationships of deep eutectic solvents during rice straw pretreatment. Bioresour Technol 249:261–267. 10.1016/j.biortech.2017.10.019 29049985 10.1016/j.biortech.2017.10.019

[45] Jančíková V, Jablonský M (2024) Exploiting deep eutectic solvent-like mixtures for fractionation biomass, and the mechanism removal of lignin: a review. Sustainability 16(2):504. 10.3390/su16020504

[46] Suopajarvi T, Ricci P, Karvonen V, Ottolina G, Liimatainen H (2020) Acidic and alkaline deep eutectic solvents in delignification and nanofibrillation of corn stalk, wheat straw, and rapeseed stem residues. Ind Crops Prod 145:111960

[47] Maibam PD, Goyal A (2022) Approach to an efficient pretreatment method for rice straw by deep eutectic solvent for high saccharification efficiency. Bioresour Technol 351:127057. 10.1016/j.biortech.2022.127057 35337995 10.1016/j.biortech.2022.127057

[48] Zheng Q, Zhou T, Wang Y, Cao X, Wu S, Zhao M, Wang H, Xu M, Zheng B, Zheng J, Guan X (2018) Pretreatment of wheat straw leads to structural changes and improved enzymatic hydrolysis. Sci Rep 8:1321. 10.1038/s41598-018-19517-5 29358729 10.1038/s41598-018-19517-5 PMC5778052

[49] Chen Z, Reznicek WD, Wan C (2018) Deep eutectic solvent pretreatment enabling full utilization of switchgrass. Bioresour Technol 263:40–48. 10.1016/j.biortech.2018.04.058 29729540 10.1016/j.biortech.2018.04.058

[50] Chang KL, Liu CH, Phitsuwan P, Ratanakhanokchai K, Lin YC, Dong CD, Yang GC (2021) Enhancement of biological pretreatment on rice straw by an ionic liquid or surfactant. Catalysts 11(11):1274. 10.3390/catal11111274

[51] Sharma N, Allardyce BJ, Rajkhowa R et al (2023) Rice straw-derived cellulose: a comparative study of various pre-treatment technologies and its conversion to nanofibres. Sci Rep 13:16327. 10.1038/s41598-023-43535-7 37770522 10.1038/s41598-023-43535-7 PMC10539515

[52] Chen YW, Lee HV, Abd Hamid SB (2017) Facile production of nanostructured cellulose from *Elaeis guineensis* empty fruit bunch via one pot oxidative-hydrolysis isolation approach. Carbohydr Polym 157:1511–1524. 10.1016/j.carbpol.2016.11.030 27987863 10.1016/j.carbpol.2016.11.030

[53] Zhu Y, Qi B, Liang X, Luo J, Wan Y (2021) Comparison of corn stover pretreatments with Lewis acid catalyzed choline chloride, glycerol and choline chloride-glycerol deep eutectic solvent. Polymers 13(8):1170. 10.3390/polym13071170 33917314 10.3390/polym13071170 PMC8038657

[54] Kumar AK, Parikh BS, Pravakar M (2016) Natural deep eutectic solvent mediated pretreatment of rice straw: bioanalytical characterization of lignin extract and enzymatic hydrolysis of pretreated biomass residue. Environ Sci Pollut Res 23(10):9265–9275. 10.1007/s11356-015-4780-4 10.1007/s11356-015-4780-4 26032452

[55] Hsu TC, Gou GL, Chen WH, Hwang WS (2010) Effect of acid pretreatment of rice straw on structural properties and enzymatic hydrolysis. Bioresour Technol 101:4907–4913. 10.1016/j.biortech.2009.10.009 10.1016/j.biortech.2009.10.009 19926476

[56] Ma H, Fu P, Zhao J, Lin X, Wu W, Yu Z, Zhou J (2022) Pretreatment of wheat straw lignocelluloses by deep eutectic solvent for lignin extraction. Molecules 27(22):7955. 10.3390/molecules27227955 36432056 10.3390/molecules27227955 PMC9697946

[57] Igbojionu LI, Laluce C (2023) Optimization of high-solid enzymatic hydrolysis of two-step alkaline and dilute acid-pretreated sugarcane bagasse at low enzyme loadings by response surface methodology. Biomass Convers Biorefin 13(7):5821–5830. 10.1007/s13399-021-01544-4

[58] Chen N, Jiang K, Zhao M, Zhang C, Jin Y, Wu W (2024) Pretreatment process of lignocellulosic biomass: a review of pseudo-lignin formation. Biomass Bioenerg 188:107339. 10.1016/j.biombioe.2024.107339

[59] El-Sayed SA, Khass TM, Mostafa ME (2024) Thermal degradation behaviour and chemical kinetic characteristics of biomass pyrolysis using TG/DTG/DTA techniques. Biomass Conv Biorefin 14:17779–17803. 10.1007/s13399-023-03926-2

[60] Almazrouei M, Adeyemi I, Janajreh I (2022) Thermogravimetric assessment of the thermal degradation during combustion of crude and pure glycerol. Biomass Convers Biorefin 12(10):4403–4417. 10.1007/s13399-022-02526-w

[61] Wang Z, McDonald AG, Westerhof RJ, Kersten SR, Cuba-Torres CM, Ha S, Garcia-Perez M (2013) Effect of cellulose crystallinity on the formation of a liquid intermediate and on product distribution during pyrolysis. J Anal Appl Pyrolysis 100:56–66. 10.1016/j.jaap.2012.11.017

[62] Dev B, Bakshi A, Paramasivan B (2022) Prospects of utilizing seawater as a reaction medium for pretreatment and saccharification of rice straw. Chemosphere 293:133528. 10.1016/j.chemosphere.2022.133528 34995624 10.1016/j.chemosphere.2022.133528

[63] Yu Y, Wang D, Chen L, Qi H, Liu A, Deng M, Wang K (2022) Recyclable choline chloride-based deep eutectic solvent pretreatment for accelerated enzymatic digestibility of *Triarrhena lutarioriparia*. Ind Crops Prod 187:115542. 10.1016/j.indcrop.2022.115542

